# Rational Framework for the Design of Trp- and Arg-Rich Peptide Antibiotics Against Multidrug-Resistant Bacteria

**DOI:** 10.3389/fmicb.2022.889791

**Published:** 2022-05-23

**Authors:** Wenyu Xiang, Patrice Clemenza, Jessie Klousnitzer, Jespar Chen, Weiheng Qin, Stephanie Tristram-Nagle, Yohei Doi, Y. Peter Di, Berthony Deslouches

**Affiliations:** ^1^Department of Environmental and Occupational Health, School of Public Health, University of Pittsburgh, Pittsburgh, PA, United States; ^2^Biological Physics Group, Department of Physics, Carnegie Mellon University, Pittsburgh, PA, United States; ^3^Division of Infectious Diseases, University of Pittsburgh School of Medicine, Pittsburgh, PA, United States

**Keywords:** antimicrobial peptides, antibiotic resistance, peptide antibiotics, multidrug resistance, ESKAPE pathogens, antimicrobial agents, cationic amphipathic peptides, cationic polymers

## Abstract

The threat of antibiotic resistance warrants the discovery of agents with novel antimicrobial mechanisms. Antimicrobial peptides (AMPs) directly disrupting bacterial membranes may overcome resistance to traditional antibiotics. AMP development for clinical use has been mostly limited to topical application to date. We developed a rational framework for systematically addressing this challenge using libraries composed of 86 novel Trp- and Arg-rich engineered peptides tested against clinical strains of the most common multidrug-resistant bacteria known as ESKAPE pathogens. Structure-function correlations revealed minimum lengths (as low as 16 residues) and Trp positioning for maximum antibacterial potency with mean minimum inhibitory concentration (MIC) of 2–4 μM and corresponding negligible toxicity to mammalian cells. Twelve peptides were selected based on broad-spectrum activity against both gram-negative and -positive bacteria and <25% toxicity to mammalian cells at maximum test concentrations. Most of the selected PAX remained active against the colistin-resistant clinical strains. Of the selected peptides, the shortest (the 16-residue E35) was further investigated for antibacterial mechanism and proof-of-concept *in vivo* efficacy. E35 killed an extensively-resistant isolate of *Pseudomonas aeruginosa* (PA239 from the CDC, also resistant to colistin) by irreversibly disrupting the cell membranes as shown by propidium iodide incorporation, using flow cytometry and live cell imaging. As proof of concept, *in vivo* toxicity studies showed that mice tolerated a systemic dose of up to 30 mg/kg peptide and were protected with a single 5 mg/kg intravenous (IV) dose against an otherwise lethal intraperitoneal injection of PA239. Efficacy was also demonstrated in an immune-compromised Klebsiella pneumoniae infection model using a daily dose of 4mg/kg E35 systemically for 2 days. This framework defines the determinants of efficacy of helical AMPs composed of only cationic and hydrophobic amino acids and provides a path for a potential departure from the restriction to topical use of AMPs toward systemic application.

## Introduction

The membrane-perturbing mechanism of many antimicrobial peptides makes an appealing case for their development as a potentially effective therapeutic source against infections associated with multidrug-resistant (MDR) bacteria (Casciaro et al., 2017; Magana et al., 2020). The magnitude of the crisis constituted by bacterial resistance to traditional antibiotics has elicited an immense effort to investigate such a diverse class of agents (de Breij et al., 2018; Mwangi et al., 2019), despite persistent biases against further antimicrobial peptide (AMP) development incited by the initial shortcomings of AMPs. Since their discoveries as the first line of the innate defense system against infectious pathogens over four decades ago, AMPs have been the subject of a vast literature, revealing thousands of natural AMPs across most life forms and extensive data on structure-function correlations (Bishop et al., 2017; Zouhir et al., 2017). Despite all these efforts, most classically synthesized AMPs, which are made with conventional amino acids, have failed some of the rigorous tests in advanced phases of clinical development. In addition, clinical development is typically directed toward local delivery to the sites of infection (de Breij et al., 2018; Jiang et al., 2019; Mourtada et al., 2019; Mwangi et al., 2019).

The lack of clinical evidence for systemic use after decades of research often leaves the AMP field with a sense that the expectations from the promising data on AMPs may never come to fruition. One apparent reason is that the AMP field only advances incrementally. In addition, while many discoveries seem promising, these advances often lead to scattered information that is sometimes repetitive without resulting in specific and definitive structure-function correlations, which would reduce the need for trial and error in AMP design and structural optimization. Nevertheless, the lack of clinical evidence for systemic *in vivo* efficacy of classical AMPs should not undermine the pioneering works on AMP mechanisms and structural optimization (Martin et al., 1995; Rozek et al., 2000; Lipsky et al., 2008; Nguyen et al., 2011). In particular, studies of Trp (W)-rich peptides using *de novo*-engineered (e.g., WLBU2) as well as natural and host-derived AMPs such as tritrpticin, indolicidin, LL37-derived SAAP-148 and ZY4 have led to the recognition of the therapeutic potential of W-based peptides in enhancing the antibacterial potency of AMPs (Deslouches et al., 2005b; Mwangi et al., 2019; Di et al., 2020). In in the last two decades, some of these studies have resulted in a conceptual shift from *in vitro* structure-function studies to proof of concept using small animal treatment models, with several AMPs now in clinical trials, including LL37 and WLBU2 (PLG0206) (Deslouches et al., 2007; Greber and Dawgul, 2017; Dijksteel et al., 2021). However, most of these *in vivo* efficacy studies tend to focus on topical use or local delivery of the peptides to the site of administration of the bacterial inoculum (de Breij et al., 2018; Di et al., 2020).

Another paramount challenge in AMP design is to increase antibacterial potency without markedly increasing the risk of host toxicity. This is partly due to varying degrees of trial and error in current approaches to AMP design. In this context, our central hypothesis is that antibacterial activity and toxicity to mammalian cells or host organisms can be effectively uncoupled by the complete dissection of the cationic amphipathic motif based on well-controlled differences in W content and positioning, length, charge, and hydrophobic residues. To address our hypothesis, we drew lessons from the fact that many structure-function studies using different host-derived AMP templates (e.g., SAAP-148 derived from LL37) do not necessarily result in generalized structure-function correlations partly because of the diversity in amino acid composition of such templates (de Breij et al., 2018). A plausible explanation is that these natural AMP templates already include amino acids that may not determine antimicrobial functions, as they are not typically hydrophobic or cationic. Instead, their presence in the primary sequences of these natural peptides may be associated with the multifunctionality of AMPs and influence the folding of the primary sequence in a way that may partially hinder optimization specific to antimicrobial function (Kumar et al., 2018; Levast et al., 2019). From an antimicrobial standpoint, we needed to control for these factors. To do so, we only used amino acids that are typically cationic or hydrophobic, which are more relevant to AMP amphipathic structure as determinants of antimicrobial properties. In addition, it was important to explore the well-studied role of W in overcoming many AMP limitations such as susceptibility to salt and serum as well as the lack of *in vivo* efficacy (Deslouches et al., 2020). Further, we retained Arg (R) over Lys because it is the most basic amino acid and shown to be effective in previous studies. It is in this context that we report herein a systematic assessment of the impact of W on selective potency against the most common MDR bacteria known as ESKAPE pathogens (Boucher et al., 2009; Deslouches et al., 2015). Toward this goal, we engineered (*de novo*) multiple W-based libraries of cationic peptide antibiotics (PAX, designated as a distinction from natural and other engineered AMPs), consisting of a total of 86 peptides in addition to several AMP and antibiotic controls. Our study elucidates measurable functional changes associated with minor structural differences to rationally achieve selective antibacterial properties in a way that does not appear to critically affect host toxicity. *In vitro* structure-function correlations resulted in rationally selected PAX candidates that displayed broad efficacy against MDR strains of ESKAPE pathogens, including antibiotic-resistant isolates (AR isolates) from the Center for Disease Control and Prevention (CDC). As proof of concept, one of the selected PAX was tested systemically and demonstrated the ability to mitigate an otherwise lethal infection induced by an MDR strain of *Pseudomonas aeruginosa* in mice.

## Materials and Methods

### Reagents, Bacteria, and Antibacterial Assays

Bacterial media cation-adjusted Mueller Hinton Broth (MHB2) was obtained from Millipore Sigma (St Louis, MO, United States). Fixable live/dead stain and Propidium iodide (PI) were purchased from Thermo Fisher Scientific (Waltham, MA United States). All peptides were obtained in lyophilized form of 10 mg in a 1.5 mL vial from Genscript (NJ, United States) with HPLC/MS spectra corresponding to each designed primary sequence. Each peptide vial was labeled with a code name (E followed by a number) without sequence identity, including peptide controls (e.g., indolicidin) (Nielsen et al., 2019), and was dissolved by adding 1 mL filter-sterilized PBS to each peptide (10 mg/mL). The comprehensive names, which were used during the design of libraries, were only utilized after the analysis of the data. The traditional antibiotics and colistin were all purchased from Millipore Sigma (St Louis, MO, United States).

Bacterial clinical isolates, used for initial screening and in previous studies (Deslouches et al., 2013; Lin et al., 2018), were anonymously provided by the clinical microbiology laboratory of the University of Pittsburgh medical Center. The panel used for secondary screening was from CDC (2013) (Supplementary Table 3), with resistance profiles data. Bacteria are stored in lock-secured –80°C freezer and typically retrieved by obtaining single colonies on agar plates prior for subsequent liquid broth cultures. Suspensions of test bacteria were prepared from log phase of growth by diluting overnight cultures at 1:100 with fresh cation-adjusted MHB (MHB2, Millipore Sigma, United States) and incubating for additional 3–4 h. Bacteria were spun at 3,000 × *g* for 10 min and the pellet resuspended in test condition media (Millipore Sigma, United States) to determine bacterial turbidity using a Den-1B densitometer (Grant Instruments, United States) at 0.5 McFarland (unit of bacterial density) corresponding to 108 CFU/mL.

To examine antibacterial activity, we used minor modifications of a standard growth inhibition assay endorsed by the Clinical and Laboratory Standards Institute (CLSI), as previously described (Deslouches et al., 2013). Bacteria were incubated with each of the indicated peptides in MHB2. The bacterial cells were kept in an incubator for 18 h at 37°C, which is linked to a robotic system that feeds a plate reader every hour with one of 8 × 96-well plates. This set-up allows the collection of growth kinetic data at A 570 (absorbance at 570 nm) to examine growth inhibition in real time (BioTek Instruments, United States). We defined minimum inhibitory concentration (MIC) as the minimum peptide concentration that completely prevented bacterial growth, objectively indicated a flat (horizontal line) bacterial growth kinetic curve at A570. The assays are typically repeated a second time. If the MIC is different, a third experimental trial is performed to confirm the MIC. To assess bactericidal properties of the peptide, the assay was modified by using 15% fetal bovine serum (FBS, for a more physiologically relevant test condition) mixed with 25% MHB2 and 60% PBS. Bacterial cells (colony forming unit/mL or CFU/mL) were enumerated after incubating treated bacteria for 3 h and plating serially diluted samples on broth agar medium at 37°C overnight. Minimum bactericidal concentration (MBC) was determined as the peptide concentration at which complete bacterial killing was observed. For killing kinetics, we slightly modified the bactericidal assay to examine bacterial killing at the MBC (or 2× MBC) as a function of time for up to 3 hours. Data were analyzed using GraphPad Prism software.

### Determination of Toxicity to Mammalian Cells

Toxicity to primary cells was examined using human red blood cells (RBCs) and peripheral mononuclear cells (PBMC or white blood cells) as previously described (Deslouches et al., 2016). Briefly, RBCs and WBCs were separated by histopaque differential centrifugation using blood anonymously obtained from the Central blood Bank (Pittsburgh, United States). For RBC lysis assay, the isolated RBCs were resuspended in PBS at a concentration of 5%. The peptides were serially diluted twofold in 100 μL of PBS prior to addition of 100 μL of 5% RBC to a final dilution of 2.5% RBC in order to ensure that the absorbance (570 nm) of hemoglobin does not surpass the maximum detectable absorbance of the plate reader. In parallel, the RBCs were treated with water at different concentrations to generate a standard curve of RBC lysis that was be used to determine the percent RBC lysis in the test samples. Experiments were independently conducted by three technicians to ensure reproducibility.

Human WBCs were treated with each selected peptide in RPMI and 10% FBS and incubated for 1 h at 37°C. The cells were then immediately washed with PBS at 1,000 *g* for 7 min, while in a round-bottom 96-well plate. After resuspension in PBS, we added fixable blue live/dead stain from Life Technologies according to the manufacturer’s instructions. The cells were again washed and resuspended in PBS to remove non-specific stain and then fixed with 4 % formaldehyde (Thermo Fisher Scientific) for 1 h. After washing with PBS, the samples were stored at 4°C overnight (in the dark) before examination by flow cytometry using the Novocyte flow cytometer (Agilent, United States). Peptide-treated samples were compared with untreated control for incorporation of the dye, and data were analyzed using the Novocyte analytical software. Dye incorporation was quantified as Percent toxicity directly determined by distinguishing live from dead populations (Deslouches et al., 2016), which was plotted using GraphPad (Prizm software).

### Circular Dichroism Spectroscopy and Flow Cytometry

Synthetic lipids were purchased from Avanti Polar Lipids (Alabaster, AL, United States) in the lyophilized form and used as received. Cholesterol was from Nu-Chek-Prep (Waterville, MN, United States). HPLC grade organic solvents were from Sigma-Aldrich (St. Louis, MO, United States). Lipid membrane mimics were prepared by mixing stock solutions in chloroform to achieve the following lipid:lipid molar ratios. Gram-negative inner membrane: (7:2:1) 1-palmitoy-2-oleoyl-sn-glycero-3-phosphoethanolamine (POPE): 1-palmitoyl-2-oleoyl-sn-glycero-3-phospho-(10-rac-glycerol) sodium salt (POPG): 10,30-bis-[1,2-dioleoyl-sn-glycero-3-phospho]-sn-glycerol sodium salt (TOCL, i.e., cardiolipin); Gram-positive cellular membrane: (6:1.5:1.5:1) POPG: 1,2-dioleoyl-3-trimethyl-ammoniumpropane chloride salt (DOTAP): POPE: TOCL; Euk33: (5:1:3) 1-palmitoyl-2-oleoyl-sn-glycero-3-phosphocholine (POPC):POPE: cholesterol. Lipid stock solutions were vacuum dried, then hydrated in 15 mM PBS by thermal cycling and vortexing to produce 20 mg/mL multilamellar vesicles, which were then extruded through 500 Å nucleopore filters 21 times to produce 15 mg/mL unilamellar vesicles (ULVs) (determined gravimetrically). ULVs were added to 3 mL 10 μM peptide in 15 mM PBS with pH ∼7 to produce the following lipid:peptide molar ratios: 5:1, 10:1, 20:1, 30:1, 50:1, 70:1. Data were collected in quartz cuvettes with a 1 cm pathlength using a Jasco 1500 circular dichroism (CD) spectrometer. Samples were allowed to equilibrate for 5 min at 37°C, and then scanned from 200 to 240 nm 20 times and the results were averaged. The parameters for scanning were at a speed of 100 nm/min, a step size of 1.0 nm, a response time of 1 s, a bandwidth of 1 nm, and a sensitivity of 20 mdeg. Background scans of the same concentration of ULVs were first subtracted from the raw ellipticity traces; sometimes traces were smoothed using adjacent averaging of 2–5 nm. OriginPro was used to carry out a linear least squares fit of the ellipticity traces to four secondary structural motifs representing α-helix, β-sheet, β-turn, and random coil (Heinrich et al., 2020). This analysis gives a percentage match of each of the secondary structural motifs to the total sample ellipticity. Goodness of fit was evaluated visually and quantitated using an adjusted R2. Various constraints and weighting procedures were used to optimize the fits.

To examine whether the selected peptides kill their bacterial target mainly by membrane permeabilization, we used PI incorporation by the MDR strain PA239 (5 × 10^8^ CFU/mL) quantitated by flow cytometry (Novocyte, United States) or visualized by video imaging using the digital microscope EVOS M7000 (Thermo Fisher Scientific, United States) in bright field or red fluorescence channel. In the flow cytometry experiments (Di et al., 2020), bacterial cells were either untreated or treated with different peptide E35 concentrations in MHB2 for 1 h at 37°C. We washed the bacteria-peptide mixture with PBS using an Eppendorf centrifuge at top speed for 7 min and incubated with PI in the dark for 15 min at room temperature. Tobramycin was used as a negative control with a 3-h long incubation. For video imaging, the broth medium was reduced to 20% in PBS (treated or untreated) to decrease the rate of cell division and PI incorporation assessed over time. Video images were recorded in real time using the 40× objective either in bright field or red fluorescence channel.

### Mouse Toxicity and Infection Treatment Models

For all animal experiments, we used the protocol #18071776 approved by the Institutional Animal Care and Use Committee (IACUC) of the University of Pittsburgh, which is consistent with the NIH guide for the care and use of laboratory animals. In a preliminary (pilot) experiment, female CD-1 mice (three per group) weighing about 20 g were injected different doses of peptides up to 45mpk *via* a lateral tail vein (IV) to determine an MTD (dose that results in no apparent morbidity or mortality). To confirm the MTD determined in the pilot study, CD-1 mice (seven per group) were randomized to receive a dose based on the preliminary MTD (IV) or PBS (100 μL). The animals were monitored for up to 7 days for morbidity and survival.

Three to four-week old female CD-1 mice (<20 g) were purchased from Charles Rivers. *P. aeruginosa* infection was established by intraperitoneal (IP) injection of 1 to 5 × 10^7^ bacterial CFU in a small pilot experiment to determine a minimum bacterial lethal dose (MBLD, bacterial dose resulting in 100% mortality). The MBLD was then used to induce infection in our treatment model. The bacterial inoculum was confirmed by serial dilution and plating on agar of a 0.5 McFarland bacterial suspension followed by incubation at 37°C overnight. Infected mice were typically treated with PBS or peptide 1-2 h after bacterial exposure. A peptide dose of 4–5mg/kg was used in 100 μL PBS. We also used an immunocompromised mouse model using cyclophosphamide injection, 150 mg/kg on first injection and 120 mg/kg daily for four consecutive days. *K. pneumoniae* was injected i.p. on day 6, and treatment was initiated ∼2 h post-infection and continued daily for 2 days (a total of 2 doses of 4 mg/kg). During the experiments, we monitored the mice for signs of morbidity and survival or mortality (moribund state). To examine the differential bacterial burden in mock-treated and peptide-treated groups, mice were euthanized by CO_2_ inhalation at 5h post-treatment. Bacterial load in the blood and in total lung homogenates was determined by serial dilution and plating on agar overnight before enumeration as CFU/mL of blood or homogenized tissue (lung) (Di et al., 2020).

### Statistical Analysis

We typically represent the results in graph format as mean ± standard deviation displayed by the error bars. For *in vitro* studies and bacterial burden in tissues, we analyzed differences between test samples for statistical significance using unpaired t test with two-tailed P values or the Kolmogorov-Smirnov test. For survival studies, we used Kaplan–Meir analysis with the Log-rank (Mantel–Cox) and Gehan–Breslow–Wilcoxon tests. The error bars in the CD analysis were obtained from several fitting attempts of a single sample, as well as from duplicate samples. We considered *P* values under 0.05 for statistical significance. The GraphPad software was used for all analyses unless otherwise indicated.

## Results

### Rational Framework for Engineering W-Rich Peptide Antibiotics With Enhanced Selectivity Against Drug-Resistant Bacteria

**FIGURE 1:**
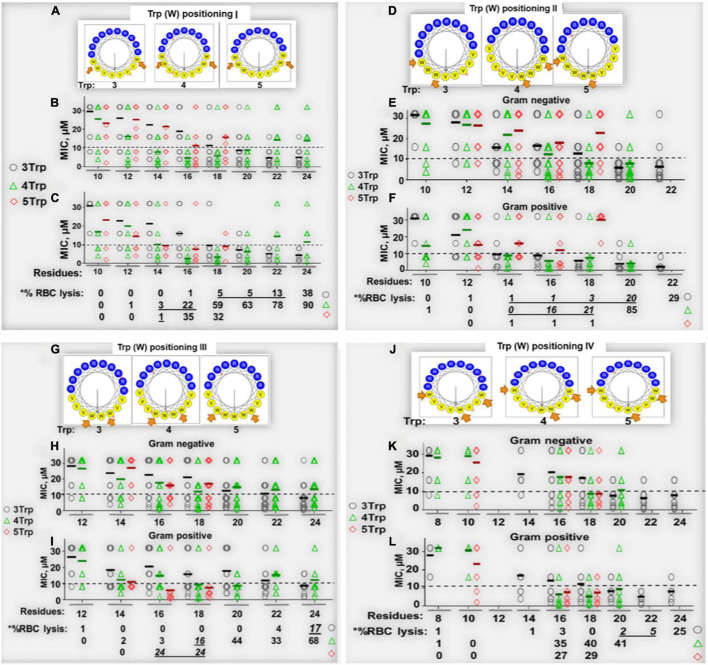
Determinants of selectivity of engineered Trp-rich cationic peptide antibiotics (PAX). Shown in panels **(A,D,G,J)** are helical wheels of 18-residue PAX representing Trp positional groups I–IV, respectively, from which primary sequences were deduced and shown in Tables 1–4. The antibacterial [MIC in μM, **(B,C,E,F,H,I,K,L)**] and erythrolytic effects of PAX at the maximum test concentration of 32 μM [lower sections of panels (C,F,I,L)] are shown as a function of length in the context of Trp content (black, 3W; green, 4W; red, 5W limited to 18 residues in length). Underlined% lysis (black bars) indicate MIC < 10 μM corresponding to RBC lysis <25% at 32 μM peptide. The results are representative of 2–3 independent experimental trials.

Our study began with the design of a series of AMPs based on differential numbers of W residues (e.g., 3, 4, or 5 W residues, Table 1), charge, and peptide length. We know from past studies that the exclusive use of W in the hydrophobic domain may enhance both antimicrobial functions and host toxicity due to its high hydrophobicity and its bulky indole ring (Deslouches et al., 2005b, 2020). Thus, to balance the non-selective membrane-seeking property of W, we designated Val (V) instead of Phe, Leu, or Ile to complete the hydrophobic domain because of its substantially lower hydrophobicity than that of W. Depending on the number of W residues, we started with a minimum length of 8 (4R + 4W) or 10 (5R + 5W or 6R + 3W + 1V) residues (Table 1) using helical wheel projections to deduce the primary sequences as idealized helices with high hydrophobic moments, which is a measure of helical amphipathicity (Figure 1A; Gautier et al., 2008). We incrementally increased the length and charge of the peptides by adding 1R and 1V at a time to each template until a maximum length of 24 (3W and 4W templates) or 18 (5W template) residues was reached. The 5-W template was limited to 18 residues in length based on previous observations that increasing the number of W in longer peptides (e.g., 24 residues) markedly increased host toxicity (Deslouches et al., 2005b, 2013). In that regard, our goal was to develop a framework allowing the identification of highly potent and safe peptides that are as short as possible.

**TABLE 1:** Primary sequences and physicochemical properties of Library 1 peptides, which correspond to W-position I shown in Figures 1A–C.

Name	Trp (W)	Sequence	Length (r)	Charge*^a^*	H	μH
E23	3	WW RR VR RR WR	10	6	0.19	0.859
E19	3	WW RR VR RR WR RV	12	7	0.18	0.859
E16	3	WW RR VR RV WR RV RR	14	8	0.17	0.851
E63	3	VW RR VR RW WR RV RR	14	8	0.17	0.832
E64	3	VW RR VR RW WR RV RR RV	16	9	0.16	0.79
E13	3	WW RR VR RV WR RV RR RV	16	9	0.16	0.808
E66	3	VW RR VR RV WR RV RR WV RR	18	10	0.15	0.78
E10	3	WW RR VR RV WR RV RR VV RR	18	10	0.15	0.811
E7	3	RW VR RV RR VW RR VR RV VR RW	20	11	0.15	0.84
E72	3	RR VW RR VR RV WR RV RR WV RR VV	22	12	0.14	0.765
E4	3	RR WV RR VR RV WR RV RR VV RR WV	22	12	0.14	0.812
E76	3	RR VW RR VR RV VR RV WR WV RR VV RR	24	13	0.14	0.739
E29	4	RR WW RR VW RW	10	5	0.52	0.83
E30	4	RR VW RW VR RW WR	12	6	0.45	0.87
E21	4	WW RR WR RR WR RV	12	7	0.26	0.942
E32	4	RR VW RW VR RW WR RV	14	7	0.4	0.849
E35	4	RR VW RW VR RV WR WV RR	16	8	0.36	0.736
E38	4	RR VW RW VR RV WR RV RR WV	18	9	0.33	0.791
E41	4	RR VW RW VR RV WR WV RR VV RR	20	10	0.31	0.729
E44	4	RR VW RW VR RV WR WV RR VV RR VR	22	11	0.29	0.718
E45	4	RR VR RW VR RV WR WV RR VW RR VR	22	12	0.19	0.762
E48	4	RR VW RW VR RV WR WV RR VV RR VR RV	24	12	0.28	0.72
E49	4	RR VR RW VR RV WR WV RR VW RR VR RV	24	13	0.18	0.764
E83	5	RR WW RR WW RW	10	5	0.62	0.931
E84	5	RR WW RW VR RW WR	12	6	0.53	0.951
E86	5	RR WW RW VR RW WR RV	14	7	0.47	0.916
E89	5	RR VW RW WR RV WR WV RR	16	8	0.43	0.793
E94	5	RR VW RW VR RV WR RV RR WW	18	9	0.39	0.835

*H, hydrophobicity, and μH, hydrophobic moment, were determined using the online peptide modeling software heliquest.ipmc.cnrs.fr/; peptides of the same length are minor positional variants of W; r, residues. The sequences were modeled as idealized amphipathic helices based on helical wheel diagram using the online modeling software Heliquest, which also provides the physicochemical properties listed in the table. ^a^All charges are positive.*

To control for the potential influence of the positions of W residues, we used W-positional analysis to complement the first PAX library shown in Table 1 with three additional libraries (Tables 2–4), resulting in a total number of 86 *de novo*-engineered PAX. We distinguished them as libraries 1, 2, 3, and 4. In library 1, the W residues are placed on both sides of the hydrophobic-hydrophilic interface of the helical wheel projections of the peptides (Figure 1A). In library 2, the W residues were asymmetrically aggregated at the hydrophilic-hydrophobic interface (Figure 1D and Table 2). Library 3 was generated by placing the W residues as far as possible from the hydrophilic domain of the helical wheel projections (Figure 1G and Table 3). In library 4, the W residues are staggered or equidistantly scattered throughout the hydrophobic domain (Figure 1J and Table 4). Of note, W positions are restricted by the number of W residues and the length of each PAX. Further, there are minor positional variants of PAX of the same length in a particular library (Tables 1–4).

**TABLE 2:** Primary sequences and physicochemical properties of Library 2 peptides, which correspond to W-position II shown in Figures 1D–F.

Name	Trp (W)	Sequence	Length (r)	Charge*^a^*	H	μH
E24	3	VW RR WR RR WR	10	6	0.191	0.901
E61	3	WV RR WR RR VR RW	12	7	0.177	0.89
E62	3	VW RR WR RV WR RV RR	14	8	0.166	0.868
E65	3	VW RR VR RV WR RV RR RW	16	9	0.159	0.827
E67	3	VW RR VR RV WR RV RR VW RR	18	10	0.153	0.818
E68	3	RW VR RV RR WV RR VR RW VR RV	20	11	0.148	0.82
E69	3	RV WR RV RR VW RR VR RV WR RV	20	11	0.148	0.822
E70	3	RW VR RV RR WV RR VR RV VR RW	20	11	0.148	0.844
E71	3	RR VW RR VR RV WR RV RR VV RR VW	22	12	0.144	0.777
E73	3	RR WV RR VR RW VR RV RR VV RR WV	22	12	0.144	0.814
E74	3	RR WV RR VR RW VR RV RR WV RR VV	22	12	0.144	0.791
E75	3	RR VW RR VR RV WR RV RR VW RR VV	22	12	0.144	0.798
E77	4	RR WW RR WV RW	10	5	0.517	0.938
E31	4	RR WW RV WR RV WR	12	6	0.448	0.963
E78	4	VW RR WR RR WR RW	12	7	0.263	0.963
E79	4	RR WV RW VR RW VR RW	14	7	0.399	0.942
E80	4	RR WV RW VR RW VR WV RR	16	8	0.363	0.85
E81	4	RR WV RW VR RW VR RV RR WV	18	9	0.334	0.365
E82	4	RR VW RV WR RV WR VV RR VW RR	20	10	0.311	0.811
E85	5	RR WW RV WR RW WR	12	6	0.534	1.021
E88	5	RR WV RW WR RW VR RW	14	7	0.473	1.009
E93	5	RR WV RW VR RW VR WW RR	16	8	0.427	0.911
E95	5	RR VW RV WR RV WR RW RR VW	18	9	0.391	0.921

*H, hydrophobicity and μH, hydrophobic moment were determined using the online peptide modeling software heliquest.ipmc.cnrs.fr/; peptides of the same length are minor positional variants of W; r, residues*

*The sequences were modeled as idealized amphipathic helices based on helical wheel diagrams using the online modeling software Heliquest, which also provides the physicochemical properties listed in the table.*

*^a^All charges are positive.*

**TABLE 3:** Primary sequences and physicochemical properties of Library 3 peptides, which correspond to W-position III shown in Figures 1G–I.

Name	Trp (W)	Sequence	Length (r)	Charge*^a^*	H	μH
E20	3	VV RR WR RR WR RW	12	7	0.177	0.904
E18	3	WV RR WR RV VR RW RR	14	8	0.166	0.914
E15	3	VV RR WR RV VR RW RR RW	16	9	0.159	0.86
E12	3	WV RR WR RV VR RW RR VV RR	18	10	0.153	0.865
E9	3	RW VR RW RR VV RR WR RV VR RV	20	11	0.148	0.864
E6	3	RR WV RR WR RV VR RW RR VV RR VV	22	12	0.144	0.834
E3	3	RR VV RR WR RV VR RW VR VW RR VV RR	24	13	0.141	0.824
E22	4	RR WV RV WR RW WR	12	6	0.448	1.002
E34	4	RR WV RV WR RW VR RW	14	7	0.399	0.995
E33	4	RR WV RV WR RW WR RV	14	7	0.399	0.962
E37	4	RR WV RV WR RW VR VW RR	16	8	0.363	0.931
E40	4	RR WV RV WR RW VR RW RR VV	18	9	0.334	0.923
E43	4	RR WV RV WR RW VR VW RR VV RR	20	10	0.311	0.884
E46	4	RR WR RV VR RW VR VW RR WV RR VR	22	12	0.191	0.87
E47	4	RR WR RV VR RW VR VW RR VV RR WR	22	12	9.191	0.88
E50	4	RR WR RV VR RW VR VW RR VV RR WR RV	24	13	0.184	0.873
E87	5	RR WV RV WR RW WR RW	14	7	0.473	1.035
E92	5	RR WV RW WR RW VR VW RR	16	8	0.427	0.955
E96	5	RR WV RV WR RW VR RW RR VW	18	9	0.391	0.966

*H (hydrophobicity) and μH (hydrophobic moment) were determined using the online peptide modeling software heliquest.ipmc.cnrs.fr/; peptides of the same length are minor positional variants of W; r, residues.*

*The sequences were modeled as idealized amphipathic helices based on helical wheel diagrams using the online modeling software Heliquest, which also provides the physicochemical properties listed in the table.*

*^a^All charges are positive.*

**TABLE 4:** Primary sequences and physicochemical properties of Library 4 peptides, which correspond to W-position IV shown in Figures 1J–L.

Name	Trp (W)	Sequence	Length (r)	Charge*^a^*	H	μH
E60	3	WR RR WR RW	8	5	*	*
E17	3	VW RR VR RW VR RW RR	14	8	0.166	0.851
E14	3	VW RR WR RW VR RV RR RV	16	9	0.159	0.803
E11	3	VW RR VR RV VR RW RR WV RR	18	10	0.153	0.798
E8	3	RV WR RV RR VV RR WR RW VR RV	20	11	0.148	0.804
E5	3	RR VW RR VR RV VR RW RR WV RR VV	22	12	0.144	0.779
E2	3	RR VV RR WR RV VR RV WR WV RR VV RR	24	13	0.141	0.758
E28	4	RR WW RR WW	8	4	*	*
E25	4	WW RR WR RR WR	10	6	0.294	0.959
E36	4	RR WW RV VR RV VR WW RR	16	8	0.363	0.819
E39	4	RR WW RW VR RV VR RW RR VV	18	9	0.334	0.845
E42	4	RR WW RV VR RV VR WW RR VV RR	20	10	0.795	0.795
E90	5	RR WW RW VR RV VR WW RR	16	8	0.427	0.843
E91	5	RR WW RW VR RV WR WV RR	16	8	0.427	0.8
E97	5	RR VW RW VR RV WR RW RR WV	18	9	0.391	0.848
E98	5	RR WW RW WR RV VR RW RR VV	18	9	0.391	0.898

H (hydrophobicity) and μH (hydrophobic moment) were determined using the online peptide modeling software heliquest.ipmc.cnrs.fr/; peptides of the same length are minor positional variants of W; r, residues; * Too short to be analyzed by the software.

*The sequences were modeled as idealized amphipathic helices based on helical wheel diagrams using the online modeling software Heliquest, which also provides the physicochemical properties listed in the table.*

*^a^All charges are positive.*

As controls, we used W-containing AMPs such as indolicidin and tritrpticin (Rozek et al., 2000; Nguyen et al., 2011). We also included the LL37-derived SAAP-148 (de Breij et al., 2018), one of the most recently engineered AMPs with remarkably broad antibacterial activity and moderate RBC lysis at 16 μM. Hence, we have designed an internally controlled system using peptides that differ from each other in length by only two residues and by a charge of +1 as well as both engineered and natural AMPs that are rich in W content for additional controls (Supplementay Figure 1). A well-known AMP (L-K6L9) with no W content was also included (Braunstein et al., 2004) in addition to appropriate antibiotic controls including colistin, depending on the bacterial test organism. While the helical wheel projections are represented in Figure 1 only by 18-residue peptides, every single PAX longer than 8 residues was modeled as a cationic amphipathic helix using the online helical modeling software heliquest (Gautier et al., 2008).

Next, all peptides were initially screened for antibacterial potency against an MDR panel of gram-negative (GNB) and -positive (GPB) bacterial isolates classified as ESKAPE pathogens, which we previously used in published studies (Deslouches et al., 2015; Di et al., 2020). Within any series of peptides with constant W content, increasing the length resulted in a gradual decrease in mean MIC (library 1) toward optimal activity at a particular length against both GNB (Figure 1B) and GPB (Figure 1C). The MIC or minimum inhibitory concentration is the minimum drug concentration that results in no detectable bacterial growth, indicated by a horizontal growth kinetic curve (data not shown) with bacterial growth measured every hour. Mean MICs are lowest at 20-24 AA residues (r) for PAX of 3W, at 16r for PAX with 4W, and 14-18r for 5W peptides. Generally, once the minimum length achieving the lowest MIC (minimum optimal length or MOL) is reached, there was no additional gain in antibacterial activity (Figures 1B,C) with further increase in length or charge. Thus, we were able to identify a precise range of MOL against the 6 organisms composing the ESKAPE pathogens (Boucher et al., 2009) and *Escherichia coli*. This structure-MIC trend remained consistent across libraries 1–4 (Tables 1–4 and Figures 1D–L), although the MOL was both dependent on W content (within a library) and positions (across libraries). Activities were typically 2-fold lower (mean MIC, 2-fold higher) against GNB compared to GPB, although a 2-fold variation in MIC is not unusual in our growth inhibition assays. Thus, the data indicate broad activity of PAX against both GNB and GPB with consideration given to MIC < 10 μM for initial peptide selection. Typically, we consider a PAX with an MIC > 8 μM against a particular strain to be of negligible therapeutic potential against that strain. The MICs of the selected PAX and antimicrobial controls against individual strains are shown in Supplementary Table 1.

**FIGURE 2:**
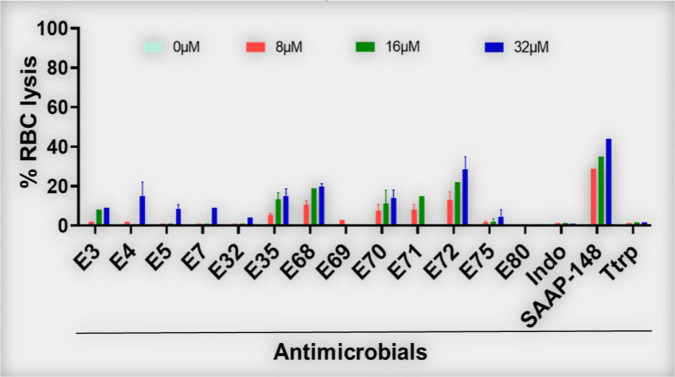
Red blood cell lytic properties of selected PAX (% RBC lysis). PAX were examined for lytic properties against freshly isolated human RBCs. Dose-dependent lytic effects of the 13 selected PAX are shown. E72 was slightly above the 25% RBC lysis cut-off. Data are representative of 2–3 experimental rials; Ttrp, tritrpticin; Ctrl-0, no agent added; SAAP, LL37-derived SAAP-148; Indo, indolicidin.

**FIGURE 3:**
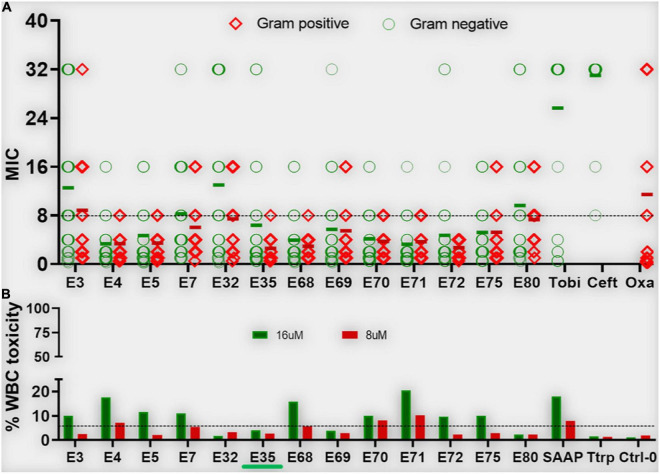
Antibacterial activity and white blood cell toxicity of selected engineered W-rich PAX. Selected PAX were examined for minimum inhibitory concentrations (MIC) **(A)** against GNB and GPB MDR isolates from the CDC shown in Supplementary Table 3. Toxicity against freshly isolated human white blood cells (WBC) **(B)** was determined by live-dead stain incorporation detected by flow cytometry. Human red blood cell lysis was determined by measuring spectrophotometrically the levels of hemoglobin release at 570 nm. Data are representative of 2–3 experimental trials. Green bar below E35 [X axis, **(B)**] indicates the PAX selected for proof-of-concept experiments; Tobi, tobramycin; Ceft, ceftazidime; Oxa, oxacillin; Ttrp, tritrpticin; Ctrl-0, no agent added; SAAP, LL37-derived SAAP-148.

Similar to structure-MIC correlations, all peptides were examined for lytic effects on RBCs as a primary measure of toxicity to mammalian cells and PAX selection. The percent RBC lysis at maximum test concentration (32 μM, Figures 1C,F,I,L, bottom panels) was shown to increase gradually with peptide length, although the minimum length required for optimal MIC usually corresponded to only minor RBC lysis (<25% at the maximum test concentration and negligible toxicity at <10 μM, Figure 2), which is an important criterion for PAX selection. This lag in RBC lysis compared to antibacterial potency at the MOL indicates the effective uncoupling of antibacterial activity and host toxicity or high antibacterial selectivity based on MIC < 10μM and RBC lysis < 25% at 32 μM. Thus, the MIC-based MOL was adjusted by considering both MIC and RBC lysis for PAX selection for advancement. That is why only twelve broadly active PAX met the selection criteria for further investigations (Figure 3). Library 1 resulted in the selection of E4 (22r in length) and E7 (20r) as 3W PAX and E32 (14r), E35 (16r) as 4W PAX; library 2: E68-70 (3W, 20r), E71 (3W, 22r), E75 (3W, 22r), E80 (4W, 16r); library 3, only E3 (3W, 24r); library 4, only E5 (3W, 22r). There was no final selection of 5W PAX, as increasing the number of W beyond 4 did not result in appreciable gain in activity compared to safety. Thus, libraries 1 (Figures 1A–C) and 2 (Figures 1D–F) produced the highest number of selected PAX (a total of 10 of the 12 selected peptides), based on mean MIC < 10μM and < 25% RBC lysis at maximum test concentration. An additional PAX (12 + 1), E72, was also included in the group of selected peptides as an example of a PAX that barely falls outside the cut-off for RBC lysis (slightly > 25%, Figure 2). Notably, colistin was inactive against the GPB strains as expected, although KPA5 is the only GNB strain to display a colistin-resistant phenotype among these initial test strains. Colistin cross-resistance of KPA5 was not observed among the selected PAX, with MIC varying from 1 to 8μM (Supplementary Table 1).

Supplementary Figure 1 showed a lack of activity of the natural AMPs indolicidin and tritrpticin (mean MIC > 10μM) against the recalcitrant panel of bacteria used in Figure 1, which is partly due to the contextual activity of these AMPs in a stringent test medium (cation-adjusted Mueller-Hinton broth, MHB2). Not surprisingly, the engineered AMP K6L9 (with no W content) was not as effective against the test bacterial panel (mean MIC > 10 μM) as the selected PAX and demonstrated a high lytic effect (> 50% at 32μM) on human RBCs. In sharp contrast, the 24-residue SAAP-148 (with 2-W content) was broadly active (mean MIC < 10μM) against both GNB and GPB, although the engineered peptide demonstrated > 40% RBC lysis at the maximum test concentration of 32μM. Notably, colistin demonstrated the lowest mean MIC, although the breakpoint for resistance is limited to approximately 2 mg/L or ∼2 μM due to its nephrotoxicity (Horcajada et al., 2016; Nation et al., 2017; Bardet and Rolain, 2018). Taken together, the data indicate that our structure-function framework constitutes a rational design system for distinguishing highly active and short peptides with minimal RBC lysis from those that are highly erythrolytic (>25% lysis at high concentrations) above the MOL.

Based on our primary structure-function correlations, we further examined the 13 selected PAX for broad antibacterial activity against MDR clinical strains of ESKAPE pathogens and *E. coli* provided by the CDC (AR isolates panel), with individual PAX MICs and resistance profiles of these strains shown in Supplementary Tables 2, 3, respectively. Similar to the primary screening, the advanced testing utilized growth inhibition assays in MHB2. Remarkably, the majority of the selected PAX remained broadly active against both GNB and GPB AR isolates from the CDC (mean MIC < 10μM for most selected PAX, Figure 3A) with slightly lower mean MICs against GPB, consistent with our initial observation with the ESKAPE panel shown in Figure 1. As a progression from the primary screening, toxicity to freshly isolated human white blood cells (WBC) was also examined to further select for lower toxicity among these broadly active peptides. The selected PAX at a maximum test concentration of 16μM displayed a toxicity level against WBC (measured by flow cytometry) that was consistently low or negligible (typically <10%) (Figure 3B). Unlike the first MDR panel shown in Supplementary Table 1, some level of cross-resistance was observed among 8 PAX for KP542 and 5 PAX for PA229 (MIC > 16 μM) (Supplementary Table 2). To further investigate cross-resistance to colistin, we identified several additional colistin-resistant KP strains according to CDC data for comparison of a subset (5) of the selected PAX. Interestingly, the select PAX displayed MICs in the range of 1–8 μM (Table 5). The differential activities indicate that, in contrast to the two cases of cross-resistance with colistin (Supplementary Table 2), resistance to colistin can be overcome by structurally optimized PAX. In that context, a PAX cocktail may be an effective option to obtain broad coverage against extensively resistant GNB isolates that are also recalcitrant to colistin treatment. Importantly, PAX activity extends across both GNB and GPB, in contrast to colistin activity only against GNB. Next, we chose a single PAX and an extensively resistant bacterial strain (PA239, Supplementary Table 3) to conclude the current investigation with mechanism and proof-of-concept experiments. Similar to the other selected PAX, E35 displayed broad activity against both test panels of GNB and GPB (Figures 1, 3) and remained active against several colistin-resistant strains. We ultimately chose E35 because it displayed the shortest MOL (at 16r), as length was an important structural parameter in our structure-function studies. However, we recognize that any of the remaining selected PAX may display a lower MIC than that of E35 against specific strains or vice versa.

**TABLE 5:** Select PAX activity against colistin-resistant CDC KP isolates, not included in Figures 1, 2.

			*MIC, μM			
	E4	E5	E35	E72	E70	colistin
KP1010	4	2	8	1	1	16
KP87	4	2	8	1	1	>16
KP507	4	4	4	4	2	16
KP1045	4	4	8	4	2	>16
KP1041	4	4	2	4	4	>16
KP46	2	2	4	2	2	4
KP125	2	2	2	2	8	8

*Break point for resistance to colistin is 2 μg/mL or ∼2 μM (Nation et al., 2017; Ooi et al., 2019); MIC >8 μM, PAX resistance; KP, Klebsiella pneumoniae.

### PAX E35 Kills *P. aeruginosa* by Perturbing the Bacterial Membrane

**FIGURE 4:**
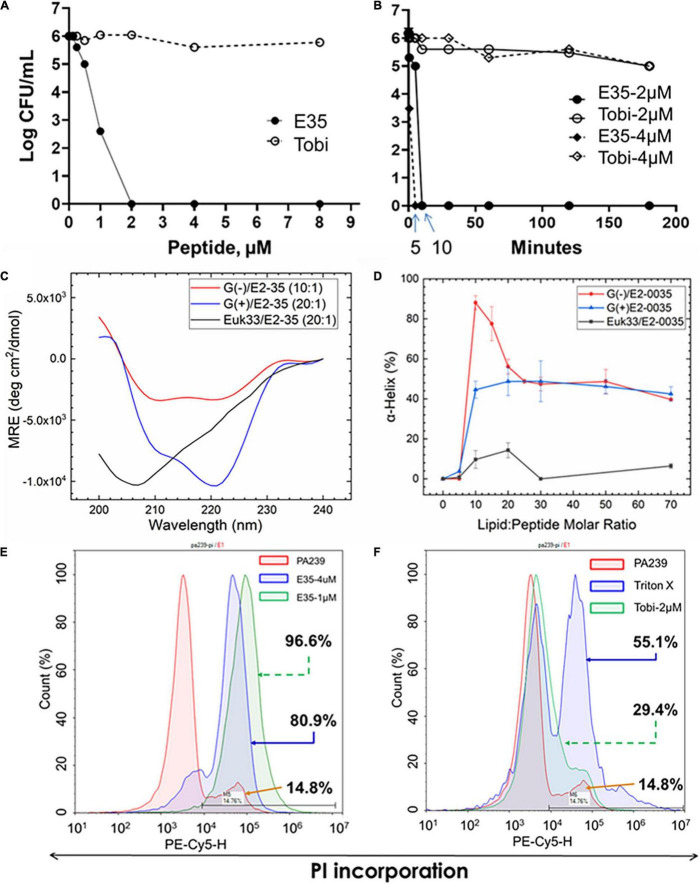
Selective killing mechanism. PAX E35 of 16 residues (Figure 1 and Table 1) was first tested for bactericidal activity and killing kinetics against *P. aeruginosa* strain PA239 (Supplementary Table 3) in fetal bovine serum containing medium **(A,B)**, with tobramycin as control. E35 interaction with membrane mimics was characterized by circular dichroism **(C,D)**. Finally, Membrane perturbation was examined by propidium Iodide incorporation detected by flow cytometry using PA239 treated with E35 **(E)** and tobramycin **(F)**. Data are representative of two independent experimental trials. Membrane mimics: G(+), gram positive; (Beringer et al., 2016), gram negative; Euk, eukaryotic.

While multiple antibacterial mechanisms have been demonstrated for a variety of cationic AMPs (Brogden, 2005; Bechinger and Gorr, 2017), membrane perturbation is the most predominant mechanism of helical AMPs (Lohner, 2017; Roncevic et al., 2019). To test whether PAX follow this common mechanism, we initially examined the bactericidal activity of the top selection, PAX E35, against the MDR *P*. *aeruginosa* strain PA239 (resistant to all 12 test antibiotics, including colistin, Supplementary Table 3). We sought to assess bactericidal properties using a medium called FMP (FBS, 15%; MHB2, 25%; PBS, 60%) that is more physiologically relevant than MHB2 with the inclusion of saline and fetal bovine serum, a known inhibitory condition for many AMPs (Deslouches et al., 2005a; Di et al., 2020). As predicted, the peptide killed all bacterial cells at 2 μM concentration, whereas tobramycin did not display any substantial bacterial killing within 3h of incubation as expected (Figure 4A). In addition, E35 killed 2 logs of the bacterial cells within the first minute of treatment at 2 and 4 μM, compared to the non-effective tobramycin (Figure 4B). No bacterial cells survived E35 treatment after 5–10 min of incubation. However, taken together with real-time video imaging, what appeared to be complete bacterial killing within 5–10 min (Figure 4B) was partly irreversible membrane disruption that eventually resulted in cell death. Consistent with these bactericidal assays, during early exposure to the peptide PA239 at high inoculum (5 × 10^8^ CFU/mL) and 8 μM concentration, the bacterial cells displayed substantial incorporation of PI, which is impermeable to intact cell membrane. In fact, during the first 15 min (Movie 1A) of exposure to E35, bacterial cells appeared to be disintegrated as indicated by patches of DNA-containing subcellular particle aggregates. Also noteworthy was the presence of many PI-incorporated cells that retained swimming motility (Movies 1B,C). These bacterial cells continued to swim even after more than an hour (Movie 1C), indicating that a substantial number of the E35-exposed bacterial cells were enumerated as dead in the killing kinetic experiment within 5–10 min only because their cell membranes were irreversibly damaged by the peptide and, therefore, could not survive on an agar plate overnight. Certainly, many E35-treated cells died within minutes as shown by scattered aggregates of dead cells by 15 min (Video 1A). Thus, despite massive PI incorporation, real-time video imaging in bright field showed a substantial proportion of the PI-incorporated PA239 cells were still alive (swimming motility, Movies 1A–C) after 1hour of peptide treatment. In sharp contrast to E35-treated cells, mock-treated and tobramycin-treated PA239 cells displaying swimming motility (bright field, Movies 2A–D) were not visible in the PI channel (blank screen, Movies 2E–H) because they did not demonstrate any markedly detectable intracellular PI. These results indicate that the membranes of these bacterial cells remained uncompromised despite background cell death observed from the results of bactericidal assays (e.g., Figures 4A,B).

Next, we interrogated membrane mimics of GPB, GNB, and eukaryotic cells for interactions with E35 to examine their impact on the folding of the selected PAX E35 using circular dichroism (CD) (Heinrich et al., 2020). When comparing the lipid-to-peptide molar ratio at which we observed the highest helical content (Supplementary Figure 2), the CD spectra indicate that the E35 peptide has a higher propensity to fold into a helical structure in the presence of bacterial compared to eukaryotic membrane mimics (Figures 4C,D). These results are consistent with the broad antibacterial activity of PAX compared to toxicity, or lack thereof, to mammalian cells (Figures 1–3). Surprisingly, the helicity was substantially higher for GNB compared to GPB membrane mimics, although the MICs for GPB were consistently 2-fold lower than those for GNB. Thus, the differential helicity appears partly inconsistent with the differential anti-GNB and anti-GPB activities. Of note, the membrane mimics may not accurately reflect the differences in lipids displayed by the surfaces of GNB and GPB. We also confirmed that this selective interaction with bacterial membranes resulted in killing principally by membrane perturbation, using flow cytometry to detect cell death by PI incorporation. As shown in Figure 4E, the bacterial cells incorporate 81 and 97% PI in a concentration-dependent manner, 1 and 4 μM, respectively. In sharp contrast, only 29% PI incorporation was observed when cells were treated for 3 h with tobramycin (2 μM) compared to 14% incorporation of mock-treated cells. Bacterial cells treated with 1% triton X-100 displayed 55% PI incorporation (Figures 4E,F). Importantly, these cells were treated for 3 h, which may explain more background PI incorporation compared to PI detected by real-time video imaging in cells treated with drugs for a shorter period.

### Proof of Concept: Therapeutic Efficacy in *P. aeruginosa* and *Klebsiella* Models of Infection in Mice

**FIGURE 5:**
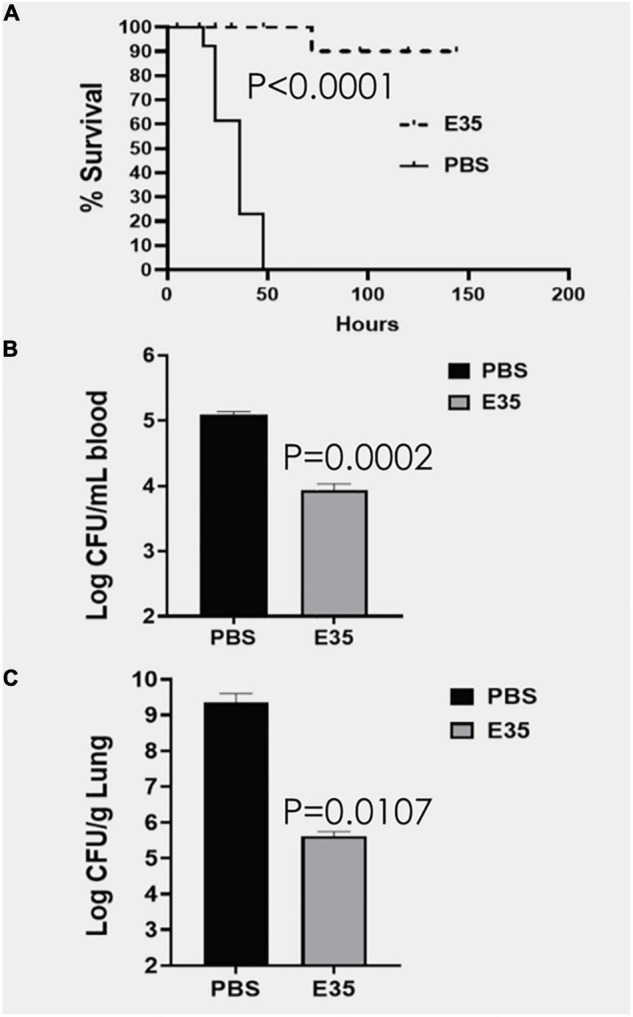
Efficacy of E35 against *P. aeruginosa* PA239. CD-1 mice (*N* = 13) were infected IP (intraperitoneally) with PA239 (∼2–3 × 10^7^ CFU) and randomly selected to receive PBS or PAX E35 at 5 mg/kg). The animals were protected from the infection, except for 1 of the 13 treated mice **(A)**. After 4 h post-treatment, bacterial burden in the blood was reduced by >10-fold **(B)**, reflective of the marked reduction of bacterial load in the lungs compared to the mock-treated mice **(C)**, consistent with the high rate of survival **(A)**. Statistical significance was determined by the Log-rank (Mantel–Cox) and Gehan-Breslow-Wilcoxon tests **(A)** as well as unpaired *t*-test with two-tailed *P* values **(B,C)**.

**FIGURE 6:**
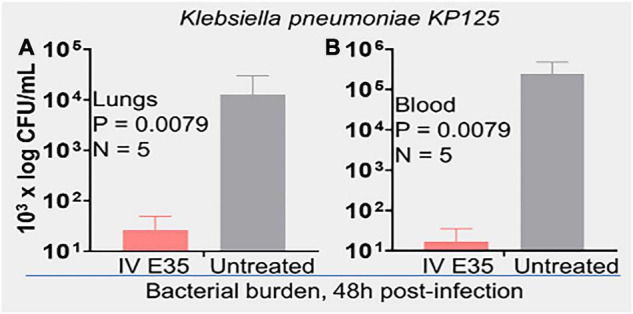
*In vivo* efficacy of PAX E35 against *K. pneumoniae* (KP125). CD-1 mice (20 g) were immunosuppressed with i.p. injection of cyclophosphamide, 150 mpk on induction and 120 mpk daily × 4 days. Infection was induced on day 6 (10^7^ CFU i.p.). Daily treatment with 4mpk IV for 2 days was initiated ∼2 h after i.p. instillation of bacteria. Bacterial load, 48 h post-infection, was determined by necropsies, and enumeration on agar plates; **(A)** lungs and **(B)** blood; statistical analysis by Kolmogorov–Smirnov test.

An important question is whether the *in vitro* activity is reflective of the therapeutic efficacy of selected peptides in animal models, which is often not the case in published studies (Dijksteel et al., 2019). To address this question, we initially sought to determine whether PAX E35 can be tolerated in mice at a high systemic dose (maximum tolerated dose, MTD). We used a pilot experiment to inject 15 CD-1 mice *via* the tail vein with various doses up to 45 mg/kg. The mice were able to tolerate the peptide at up to 30 mpk (data not shown). To confirm the MTD, 7 mice were given saline or E35 systemically at 30 mpk, and no apparent morbidity and mortality were observed (data not shown). Next, we examined PAX E35 for efficacy against PA239 in mice. We began by determining the minimum dose of PA239 lethal to CD-1 mice if injected in the intraperitoneal (IP) cavity (minimum bacterial lethal dose, MBLD). The MBLD for this MDR strain was found to be 2 × 10^7^ CFU in <20 g CD-1 mice. The mice typically succumb (100% mortality) to *P. aeruginosa* sepsis within 18–48 h (Figure 5A) in the absence of effective treatment. In sharp contrast, almost all (12/13) mice treated systemically with E35 (5 mg/kg) approximately one to two hours after bacterial exposure displayed no signs of infection when monitored up to 7 days. We examined the effects of E35 on bacterial burden at 5 h post-treatment. As shown in Figure 5B, bacterial burden in the blood was reduced by 10-fold compared to mock-treated mice. We also observed a 10,000-fold reduction in lung bacterial load (compared to mock-treated mice) (Figure 5C). Thus, using a strain that is resistant to 12 standard-of-care test antibiotics (Supplementary Table 3), including colistin, we demonstrated that the E35 peptide has the property to rapidly reduce bacterial burden within hours after the administration of a single systemic dose without affecting the ability of the animals to eliminate the remaining bacterial cells. Similar results were demonstrated when immune-compromised mice infected IP with *K. pneumoniae* (KP125, MIC in Table 1), with bacterial burden reduced by >3 logs in both the lungs (Figure 6A) and the blood (Figure 6B) after daily doses of 4 mpk of E35 for two days. Taken together with the *in vitro* studies, these results indicate that our framework can be used for elucidating the determinants of activity through structure-function correlations for the identification of PAX that are safe *in vitro* and *in vivo* and able to overcome resistance to traditional antibiotics.

## Discussion

This report highlights the development of a rational framework for the design of cationic amphipathic peptides with the property to overcome antibiotic-resistant bacterial infections. We showed that, given a *de novo* template containing W and R as the principal amino acids and varying incrementally in length by a single R (a charge of +1) and V residue at a time, it is possible to define a W content-dependent range of MOL for activity at low micromolar concentrations against MDR bacteria. The data revealed that the MOL was the lowest (16r, E35) when the W content was kept at 4 residues, and most selected peptides demonstrated moderate to negligible toxicity to freshly isolated human RBC and WBC at high test concentrations. At comparable length, the 4-W PAX series tends to display higher toxicity to mammalian cells than that of 3-W PAX. However, this toxic effect is compensated by a much shorter MOL of the 4-W at which only minor toxicity to RBC and WBC was observed at the highest test concentration, which is the key rationale for choosing E35 among the selected PAX to conclude this study. It is at the MOL that antibacterial property and toxicity to mammalian cells are typically uncoupled within the MIC range, thereby effectively enhancing the antibacterial selectivity or increasing the therapeutic index. Importantly, activity may be reduced when the peptides are elongated beyond that MOL, which is consistent with previous observations (Deslouches et al., 2005b, 2015). Additionally, the positions of the W residues may affect both antibacterial function and toxicity to host cells (Rekdal et al., 2012), which explains the differential yield in selected PAX favoring libraries 1 and 2 over libraries 3 and 4. Library 2 produced the highest number (6 of 13) of selected PAX with all W residues positioned at a single side of the hydrophobic-hydrophilic interface. Library 1 is based on the symmetric (or near-symmetric) distribution of the W residues on both sides of hydrophobic-hydrophilic interface and resulted in the selection of 5 PAX, including E35. Thus, our W-positional analysis revealed that positioning the W residues at the hydrophilic-hydrophobic interface is optimal, regardless of symmetry, for the selection of PAX that are highly potent with minor risks of host toxicity at the MOL. In our examination of toxicity, we avoided the use of immortalized cell lines to determine toxicity to host cells due to the fact that AMPs may be selectively toxic to tumor cells compared to primary cells (Wang et al., 2020; Li et al., 2021). In that context and beyond the scope of this work, our framework can also be used to selectively distinguish PAX that are highly toxic to tumor cells at concentrations that are non-toxic to primary cells, which could be a starting point for screening cationic anticancer peptides. The data also revealed evidence for the predicted bactericidal mechanism due to membrane disruption. Specifically, flow cytometry and real-time video imaging studies demonstrate that E35 killed bacteria *via* direct disruption of the membrane of the bacterial cells. Importantly, real-time video imaging was critical to the correct interpretation of the killing kinetic data, indicating that the early (5–10 min of peptide treatment) cell death was partly due to irreversibly compromised membranes of the cells, which did not growth on agar plates overnight for inclusion in the bacterial enumeration. In addition, real-time video imaging also revealed cell death during early exposure to the peptide demonstrated by the aggregates of PI-bound subcellular particles (in this case DNA). Thus, live-cell fluorescence microscopy complemented the flow data by revealing massive cellular damage (subcellular particles) that could not be detected by flow cytometry in addition to PI incorporation by live cells with compromised membranes. Of note, the high bacterial inoculum used in the mechanistic studies, compared to that used in the antibacterial assays, required a proportionally elevated PAX concentration, which was consistent with the sensitivity of the flow and video imaging.

Recent advances in the field of AMPs showed that modifications of host-derived AMP templates may result in highly potent peptides with therapeutic efficacy in mice. In particular, the LL37-derived peptides (containing 2 W residues) SAAP-148 and ZY4 demonstrate broad antibacterial activities (de Breij et al., 2018; Mwangi et al., 2019). We used SAAP-148 as one of our peptide controls because of its broad and potent activity against both GNB and GPB, which was confirmed by our preliminary screening data. However, while published data show a peptide with moderate lytic effects on the human RBCs at 16 μM, our data suggest that SAAP-148 was more erythrolytic than published data indicate. One possible explanation is that the maximum test concentration was higher in our study, and sensitivity of both studies may not be comparable. Moreover, the topical application as an ointment in mice to combat biofilm-related skin infection may not be highly relevant to RBC lysis, and the toxicity to WBC was lower than its erythrolytic effects. Another recent advance in AMP design is demonstrated by another LL37-derived peptide ZY4 (Mwangi et al., 2019). This peptide is also broadly active and highly potent against bacteria. Unlike the skin ointment application of SAAP-148, ZY4 is given IP in multiple doses using a murine IP model of bacterial infection. While the peptide was not examined for its impact on mouse survival, its effects on reducing bacterial burden were substantial and significant. Although the IP administration is certainly an important advance from the skin application of SAAP-148, similar to other examples of engineered AMPs (Mourtada et al., 2019), both bacteria and the drug were given IP and, therefore, followed the same path. Thus, it is not clear whether the efficacy of the peptide necessitated efficient absorption and distribution to tissues to suppress the infection or if the peptide acted primarily at the site of delivery. In contrast, we demonstrated that one of the selected PAX E35 works systemically against the IP-injected MDR *P. aeruginosa* by rapidly reducing the bacterial burden in blood and in lung tissue. This demonstration of efficacy is highly relevant to standard of care in hospitalized patients, who typically require IV injection to suppress MDR-related bacteremia or sepsis. The efficacy against a colistin-resistant isolate was an important consideration, although cross-resistance with E35 was observed with two (PA229 and KP542) of the 11 colistin-resistant strains. Notably, some selected PAX (e.g., E4, E68, E70, E71) displayed no cross-resistance at all, which underscores the need for an extensive comparative study of the selected PAX against MDR isolates that are highly resistant to colistin.

While we and others have previously used W, length, and charge as determinants of toxicity and antibacterial properties (Rekdal et al., 2012; Deslouches et al., 2013), this study is remarkably impactful for the following reasons. (A) It is the most systematic and extensive structure-function study of its kind utilizing 4 libraries consisting of 86 *de novo* designed peptides examined simultaneously for MIC and toxicity as a function of charge, length, as well as W content and positioning. (B) It provides a rational framework for structure-function correlations using W- and R-rich AMPs of helical tendencies. (C) Scientific rigor is indicated by only slight differences between PAX of similar W content, with the inclusion of engineered and natural AMPs in addition to standard-of-care antibiotics as controls. (D) This design system can be used as an iterative framework for testing any combination of cationic and hydrophobic amino acids or their mimics for structure-function correlations, reducing the need for trial and error. (E) The bacterial panels are highly reflective of common clinical MDR strains, using clinical isolates from our local medical center and the CDC. (F) Proof of concept was demonstrated with distinct routes of infection (IP) and treatment (systemic) of in 2 mouse infection models using 2 GNB strains (PA239 and KP125) that are colistin-resistant. This is despite that *P. aeruginosa* was not the most susceptible target (based on mean MIC) of the peptide E35 (not always the most potent of the selected PAX) among the ESKAPE pathogens, which indicates many potential applications of the selected peptides. Short-term future directions include three objectives.

(i) The selected PAX will be extensively compared for activity against a large panel of MDR bacteria that are highly resistant to colistin to better understand why the selected PAX are able to overcome colistin resistance in most cases. This will enhance our understanding of the mechanism of cross-resistance between AMPs and colistin.
(ii) The comparative studies of the selected PAX need to be extended for a deeper understanding of differential applications for more targeted clinical development.
(iii) The extensive comparison of the selected PAX will include comparative *in vivo* efficacy with exploration of the implication of the immune response in the therapeutic role of PAX.

## Conclusion

This study is an important step toward preclinical and clinical application. (1) It is a rational system for fundamental discoveries of engineered cationic peptides with enhanced safety and efficacy. (2) It is also a framework which can be extended to peptide mimics and other structural classes. Importantly, the data indicate a potential departure from topical to systemic use of engineered AMPs.

## Data Availability Statement

The original contributions presented in the study are included in the article/Supplementary Material, further inquiries can be directed to the corresponding author/s.

## Ethics Statement

The animal study was reviewed and approved by University of Pittsburgh Institutional Animal Care and Use Committee, protocol # 20026048.

## Author Contributions

WX, PC, JK, JC, and WQ performed the experiments. ST-N contributed to the study design, data interpretation, and writing of the manuscript. YD and YPD contributed to data interpretation and writing of the manuscript. BD designed the study, coordinated the project, and wrote the manuscript. All authors read and approved the final manuscript.

## Conflict of Interest

The authors declare that the research was conducted in the absence of any commercial or financial relationships that could be construed as a potential conflict of interest.

## Publisher’s Note

All claims expressed in this article are solely those of the authors and do not necessarily represent those of their affiliated organizations, or those of the publisher, the editors and the reviewers. Any product that may be evaluated in this article, or claim that may be made by its manufacturer, is not guaranteed or endorsed by the publisher.

## Supplementary Material

Supplementary Movie 1 **(A)** Real-time video imaging of PA239 treated with E35 peptide recorded after 15 min of treatment using the EVOS M7000 imager at 40× magnification in the red fluorescence (1A-15 min); only the cells that incorporate PI were visible. **(B)** Real-time video imaging of PA239 treated with E35 peptide recorded after 45 min of treatment using the EVOS M7000 imager at 40× magnification in the red fluorescence (1B-45 min); only the cells that incorporate PI were visible. **(C)** Real-time video imaging of PA239 treated with E35 peptide recorded after 70 min of treatment using the EVOS M7000 imager at 40× magnification in the red fluorescence (1C-70 min); only the cells that incorporate PI were visible. **(D)** Real-time video imaging of PA239 treated with E35 peptide recorded after 10 min of treatment using the EVOS M7000 imager at 40× magnification in the bright-field channel (1D-10 min). **(E)** Real-time video imaging of PA239 treated with E35 peptide recorded after 95 min of treatment using the EVOS M7000 imager at 40× magnification in the bright-field channel (1E-95 min).

Supplementary Movie 2 **(A)** Real-time video imaging of PA239 untreated control. The video was captured after 6 min using the EVOS M7000 imager at 40× magnification in the bright field channel (2A-6 min). **(B)** Real-time video imaging of PA239 untreated control. The video was captured after 97 min using the EVOS M7000 imager at 40× magnification in the bright field channel (2B-97 min). **(C)** Real-time video imaging of tobramycin-treated PA239. The video was captured after 35 min using the EVOS M7000 imager at 40× magnification in the bright field channel (2C-35 min). **(D)** Real-time video imaging of tobramycin-treated PA239. The video was captured after 85 min using the EVOS M7000 imager at 40× magnification in the bright field channel (2D-85 min). **(E)** Real-time video imaging of PA239 untreated control. The video was captured after 25 min using the EVOS M7000 imager at 40× magnification in the red florescence channel (2E-25 min). Only the cells that incorporated PI were visible; a blank screen indicates the absence of PI incorporation. **(F)** Real-time video imaging of PA239 untreated control. The video was captured after 100 min using the EVOS M7000 imager at 40× magnification in the red florescence channel (2F-100 min). Only the cells that incorporated PI were visible; a blank screen indicates the absence of PI incorporation. **(G)** Real-time video imaging of tobramycin-treated PA239. The video was captured after 30 min using the EVOS M7000 imager at 40× magnification in the red florescence channel (2G-30 min). Only the cells that incorporated PI were visible; a blank screen indicates the absence of PI incorporation. **(H)** Real-time video imaging of tobramycin-treated PA239. The video was captured after 80 min using the EVOS M7000 imager at 40× magnification in the red florescence channel (2H-80 min). Only the cells that incorporated PI were visible; a blank screen indicates the absence of PI incorporation.

## References

[B1] Bardet L. Rolain J. M. (2018). Development of New Tools to Detect Colistin-Resistance among *Enterobacteriaceae* Strains. *Can. J. Infect. Dis. Med. Microbiol.* 2018:3095249. 10.1155/2018/3095249 PMC6305056 30631384

[B2] Bechinger B. Gorr S. U. (2017). Antimicrobial Peptides: mechanisms of Action and Resistance. *J. Dent. Res.* 96 254–260. 10.1177/0022034516679973 27872334 PMC5298395

[B3] Beringer P. M. Bensman T. J. Ho H. Agnello M. Denovel N. Nguyen A. (2016). Rhesus theta-defensin-1 (RTD-1) exhibits in vitro and in vivo activity against cystic fibrosis strains of *Pseudomonas aeruginosa*. *J. Antimicrob. Chemother.* 71 181–188. 10.1093/jac/dkv301 26433781 PMC5007590

[B4] Bishop B. M. Juba M. L. Russo P. S. Devine M. Barksdale S. M. Scott S. (2017). Discovery of Novel Antimicrobial Peptides from Varanus komodoensis (Komodo Dragon) by Large-Scale Analyses and De-Novo-Assisted Sequencing Using Electron-Transfer Dissociation Mass Spectrometry. *J. Proteome. Res.* 16 1470–1482. 10.1021/acs.jproteome.6b00857 28164707

[B5] Boucher H. W. Talbot G. H. Bradley J. S. Edwards J. E. Gilbert D. Rice L. B. (2009). Bad bugs, no drugs: no ESKAPE! An update from the Infectious Diseases Society of America. *Clin. Infect. Dis.* 48 1–12. 10.1086/595011 19035777

[B6] Braunstein A. Papo N. Shai Y. (2004). In vitro activity and potency of an intravenously injected antimicrobial peptide and its DL amino acid analog in mice infected with bacteria. *Antimicrob. Agents. Chemother.* 48 3127–3129. 10.1128/AAC.48.8.3127-3129.2004 15273131 PMC478488

[B7] Brogden K. A. (2005). Antimicrobial peptides: pore formers or metabolic inhibitors in bacteria? *Nat. Rev. Microbiol.* 3 238–250. 10.1038/nrmicro1098 15703760

[B8] Casciaro B. Cappiello F. Cacciafesta M. Mangoni M. L. (2017). Promising Approaches to Optimize the Biological Properties of the Antimicrobial Peptide Esculentin-1a(1-21)NH2: amino Acids Substitution and Conjugation to Nanoparticles. *Front. Chem.* 5:26. 10.3389/fchem.2017.00026 PMC5404639 28487853

[B9] CDC (2013). *Antibiotic Resistance Threats in the United States, 2013.* Atlanta: Centers for Disease Control and Prevention.

[B10] de Breij A. Riool M. Cordfunke R. A. Malanovic N. De Boer L. Koning R. I. (2018). The antimicrobial peptide SAAP-148 combats drug-resistant bacteria and biofilms. *Sci. Transl. Med.* 10:eaan4044. 10.1126/scitranslmed.aan4044 29321257

[B11] Deslouches B. Gonzalez I. A. Dealmeida D. Islam K. Steele C. Montelaro R. C. (2007). De novo-derived cationic antimicrobial peptide activity in a murine model of *Pseudomonas aeruginosa* bacteraemia. *J. Antimicrob. Chemother.* 60 669–672. 10.1093/jac/dkm253 17623696 PMC3584960

[B12] Deslouches B. Hasek M. L. Craigo J. K. Steckbeck J. D. Montelaro R. C. (2016). Comparative functional properties of engineered cationic antimicrobial peptides consisting exclusively of tryptophan and either lysine or arginine. *J. Med. Microbiol.* 65 554–565. 10.1099/jmm.0.000258 27046192 PMC5042116

[B13] Deslouches B. Islam K. Craigo J. K. Paranjape S. M. Montelaro R. C. Mietzner T. A. (2005a). Activity of the de novo engineered antimicrobial peptide WLBU2 against *Pseudomonas aeruginosa* in human serum and whole blood: implications for systemic applications. *Antimicrob. Agents. Chemother.* 49 3208–3216. 10.1128/AAC.49.8.3208-3216.2005 16048927 PMC1196285

[B14] Deslouches B. Phadke S. M. Lazarevic V. Cascio M. Islam K. Montelaro R. C. (2005b). De novo generation of cationic antimicrobial peptides: influence of length and tryptophan substitution on antimicrobial activity. *Antimicrob. Agents. Chemother.* 49 316–322. 10.1128/AAC.49.1.316-322.2005 15616311 PMC538858

[B15] Deslouches B. Montelaro R. C. Urish K. L. Di Y. P. (2020). Engineered Cationic Antimicrobial Peptides (ecaps) to Combat Multidrug-Resistant Bacteria. *Pharmaceutics* 12:501. 10.3390/pharmaceutics12060501 32486228 PMC7357155

[B16] Deslouches B. Steckbeck J. D. Craigo J. K. Doi Y. Burns J. L. Montelaro R. C. (2015). Engineered cationic antimicrobial peptides to overcome multidrug resistance by ESKAPE pathogens. *Antimicrob. Agents. Chemother.* 59 1329–1333. 10.1128/AAC.03937-14 25421473 PMC4335883

[B17] Deslouches B. Steckbeck J. D. Craigo J. K. Doi Y. Mietzner T. A. Montelaro R. C. (2013). Rational design of engineered cationic antimicrobial peptides consisting exclusively of arginine and tryptophan, and their activity against multidrug-resistant pathogens. *Antimicrob. Agents. Chemother.* 57 2511–2521. 10.1128/AAC.02218-12 23507278 PMC3716171

[B18] Di Y. P. Lin Q. Chen C. Montelaro R. C. Doi Y. Deslouches B. (2020). Enhanced therapeutic index of an antimicrobial peptide in mice by increasing safety and activity against multidrug-resistant bacteria. *Sci. Adv.* 6:eaay6817. 10.1126/sciadv.aay6817 32426473 PMC7195177

[B19] Dijksteel G. S. Ulrich M. M. W. Middelkoop E. Boekema B. (2021). Review: Lessons Learned From Clinical Trials Using Antimicrobial Peptides (AMPS). *Front. Microbiol.* 12:616979. 10.3389/fmicb.2021.616979 PMC7937881 33692766

[B20] Dijksteel G. S. Ulrich M. M. W. Vlig M. Nibbering P. H. Cordfunke R. A. Drijfhout J. W. (2019). Potential factors contributing to the poor antimicrobial efficacy of SAAP-148 in a rat wound infection model. *Ann. Clin. Microbiol. Antimicrob.* 18:38. 10.1186/s12941-019-0336-7 31796055 PMC6891976

[B21] Gautier R. Douguet D. Antonny B. Drin G. (2008). Heliquest: a web server to screen sequences with specific alpha-helical properties. *Bioinformatics* 24 2101–2102. 10.1093/bioinformatics/btn392 18662927

[B22] Greber K. E. Dawgul M. (2017). Antimicrobial Peptides Under Clinical Trials. *Curr. Top. Med. Chem.* 17 620–628. 10.2174/1568026616666160713143331 27411322

[B23] Heinrich F. Salyapongse A. Kumagai A. Dupuy F. G. Shukla K. Penk A. (2020). Synergistic Biophysical Techniques Reveal Structural Mechanisms of Engineered Cationic Antimicrobial Peptides in Lipid Model Membranes. *Chemistry* 26 6247–6256. 10.1002/chem.202000212 32166806 PMC8146162

[B24] Horcajada J. P. Sorli L. Luque S. Benito N. Segura C. Campillo N. (2016). Validation of a colistin plasma concentration breakpoint as a predictor of nephrotoxicity in patients treated with colistin methanesulfonate. *Int. J. Antimicrob. Agents.* 48 725–727. 10.1016/j.ijantimicag.2016.08.020 28128096

[B25] Jiang S. Deslouches B. Chen C. Di M. E. Di Y. P. (2019). Antibacterial Properties and Efficacy of a Novel SPLUNC1-Derived Antimicrobial Peptide, alpha4-Short, in a Murine Model of Respiratory Infection. *Mbio* 10 e226–e219. 10.1128/mBio.00226-19 30967458 PMC6456746

[B26] Kumar P. Kizhakkedathu J. N. Straus S. K. (2018). Antimicrobial Peptides: diversity. *Biomolecules* 8:4. 10.3390/biom8010004 PMC5871973 29351202

[B27] Levast B. Hogan D. Van Kessel J. Strom S. Walker S. Zhu J. (2019). Synthetic Cationic Peptide IDR-1002 and Human Cathelicidin LL37 Modulate the Cell Innate Response but Differentially Impact PRRSV Replication in vitro. *Front. Vet. Sci.* 6:233. 10.3389/fvets.2019.00233 PMC6640542 31355218

[B28] Li H. Fu S. Wang Y. Yuan X. Liu L. Dong H. (2021). Antimicrobial and antitumor activity of peptidomimetics synthesized from amino acids. *Bioorg. Chem.* 106:104506. 10.1016/j.bioorg.2020.104506 33276980

[B29] Lin Q. Deslouches B. Montelaro R. C. Di Y. P. (2018). Prevention of ESKAPE pathogen biofilm formation by antimicrobial peptides WLBU2 and LL37. *Int. J. Antimicrob. Agents.* 52 667–672. 10.1016/j.ijantimicag.2018.04.019 29753132 PMC6230315

[B30] Lipsky B. A. Holroyd K. J. Zasloff M. (2008). Topical versus systemic antimicrobial therapy for treating mildly infected diabetic foot ulcers: a randomized, controlled, double-blinded, multicenter trial of pexiganan cream. *Clin. Infect. Dis.* 47 1537–1545. 10.1086/593185 18990064

[B31] Lohner K. (2017). Membrane-active Antimicrobial Peptides as Template Structures for Novel Antibiotic Agents. *Curr. Top. Med. Chem.* 17 508–519. 10.2174/1568026616666160713122404 28117020

[B32] Magana M. Pushpanathan M. Santos A. L. Leanse L. Fernandez M. Ioannidis A. (2020). The value of antimicrobial peptides in the age of resistance. *Lancet Infect. Dis.* 20 e216–e230. 10.1016/S1473-3099(20)30327-3 32653070

[B33] Martin E. Ganz T. Lehrer R. I. (1995). Defensins and other endogenous peptide antibiotics of vertebrates. *J. Leukoc. Biol.* 58 128–136. 10.1002/jlb.58.2.128 7643008

[B34] Mourtada R. Herce H. D. Yin D. J. Moroco J. A. Wales T. E. Engen J. R. (2019). Design of stapled antimicrobial peptides that are stable, nontoxic and kill antibiotic-resistant bacteria in mice. *Nat. Biotechnol.* 37 1186–1197. 10.1038/s41587-019-0222-z 31427820 PMC7437984

[B35] Mwangi J. Yin Y. Wang G. Yang M. Li Y. Zhang Z. (2019). The antimicrobial peptide ZY4 combats multidrug-resistant *Pseudomonas aeruginosa* and *Acinetobacter baumannii* infection. *Proc. Natl. Acad. Sci. U S A*. 116 26516–26522. 10.1073/pnas.1909585117 31843919 PMC6936460

[B36] Nation R. L. Garonzik S. M. Thamlikitkul V. Giamarellos-Bourboulis E. J. Forrest A. Paterson D. (2017). Dosing guidance for intravenous colistin in critically-ill patients. *Clin. Infect. Dis.* 64 565–571. 10.1093/cid/ciw839 28011614 PMC5850520

[B37] Nguyen L. T. De Boer L. Zaat S. A. Vogel H. J. (2011). Investigating the cationic side chains of the antimicrobial peptide tritrpticin: hydrogen bonding properties govern its membrane-disruptive activities. *Biochim. Biophys. Acta* 1808 2297–2303. 10.1016/j.bbamem.2011.05.015 21641334

[B38] Nielsen J. E. Lind T. K. Lone A. Gerelli Y. Hansen P. R. Jenssen H. (2019). A biophysical study of the interactions between the antimicrobial peptide indolicidin and lipid model systems. *Biochim. Biophys. Acta Biomembr.* 1861 1355–1364. 10.1016/j.bbamem.2019.04.003 30978313

[B39] Ooi M. H. Ngu S. J. Chor Y. K. Li J. Landersdorfer C. B. Nation R. L. (2019). Population Pharmacokinetics of Intravenous Colistin in Pediatric Patients: implications for the Selection of Dosage Regimens. *Clin. Infect. Dis.* 69 1962–1968. 10.1093/cid/ciz067 30722017

[B40] Rekdal O. Haug B. E. Kalaaji M. Hunter H. N. Lindin I. Israelsson I. (2012). Relative spatial positions of tryptophan and cationic residues in helical membrane-active peptides determine their cytotoxicity. *J. Biol. Chem.* 287 233–244. 10.1074/jbc.M111.279281 22057278 PMC3249074

[B41] Roncevic T. Vukicevic D. Krce L. Benincasa M. Aviani I. Maravic A. (2019). Selection and redesign for high selectivity of membrane-active antimicrobial peptides from a dedicated sequence/function database. *Biochim. Biophys. Acta Biomembr.* 1861 827–834. 10.1016/j.bbamem.2019.01.017 30710514

[B42] Rozek A. Friedrich C. L. Hancock R. E. (2000). Structure of the bovine antimicrobial peptide indolicidin bound to dodecylphosphocholine and sodium dodecyl sulfate micelles. *Biochemistry* 39 15765–15774. 10.1021/bi000714m 11123901

[B43] Wang X. Zheng Y. Bao C. Zhong G. Liu S. Wiradharma N. (2020). Branched alpha-helical peptides enhanced antitumor efficacy and selectivity. *Biomater. Sci.* 8 6387–6394. 10.1039/d0bm00629g 33029595

[B44] Zouhir A. Taieb M. Lamine M. A. Cherif A. Jridi T. Mahjoubi B. (2017). Antistaphybase: database of antimicrobial peptides (Amps) and essential oils (EOS) against methicillin-resistant Staphylococcus aureus (MRSA) and Staphylococcus aureus. *Arch. Microbiol.* 199 215–222. 10.1007/s00203-016-1293-6 27671474

